# Subharmonic instability of a self-organized granular jet

**DOI:** 10.1038/srep22520

**Published:** 2016-03-22

**Authors:** J. E. Kollmer, T. Pöschel

**Affiliations:** 1 Institute for Multiscale Simulation, Universität Erlangen-Nürnberg, Erlangen, Germany

## Abstract

Downhill flows of granular matter colliding in the lowest point of a valley, may induce a self-organized jet. By means of a quasi two-dimensional experiment where fine grained sand flows in a vertically sinusoidally agitated cylinder, we show that the emergent jet, that is, a sheet of ejecta, does not follow the frequency of agitation but reveals subharmonic response. The order of the subharmonics is a complex function of the parameters of driving.

Granular jets, that is, jets of macroscopic particles, have attracted much attention recently, mainly for their striking phenomenological similarities to fluid jets. The first similarity concerns the Rayleigh-Plateau instability^1^,^2^ breaking an narrow flow of liquid into droplets unter the action of gravity due to minimization of surface energy. Initially homogeneous granular jets falling in gravity undergo a similar instability, e.g.^3^,^4^,^5^,^6^, despite the fact that there is no surface tension. The second similarity concerns the interaction of a granular jet with a solid obstacle, e.g.^7^,^8^,^9^,^10^,^11^,^12^ where granular sheets or cone-like structures emerge. This behavior corresponds to Savart water bells observed for fluid jets^13^,^14^. The phenomenon may be described up to good accuracy by means of a vertex free, incompressible fluid flow^15^,^16^ which surprises since, in general, granular flows are neither vortex-free nor incompressible. An explanation of this coincidence and the limits of the model are given in ref. 17. Deviations from the perfect fluid description are quantified in^18^. The third similarity concern splashes which appear when a sphere is dropped into water^19^. Very similar jet-like splashes can be observed when a sphere is dropped into a loose packing of granular material^20^,^21^,^22^,^23^,^24^,^25^,^26^. Besides scientifically exciting, granular jets are important also for industrial applications such as abrasive jet micromachining^27^, jet milling^28^,^29^, erosion of granular beds when impinged by a jet^30^,^31^,^32^ and others.

In all experiments mentioned above and in most industrial applications, granular jets are generated by packing the particles into a launching tube and then shooting this plug of particles out of the pipe by means of pressurized gas, see e.g.^7^,^33^,^34^, or by similar procedures. There are, however, effects by which granular jets emerge in a self-organized way. The most prominent of them is the *oscillon*^35^ where a spatially isolated granular jet ascends from an vertically vibrated shallow granular bed each second period of the vibration. Granular jets emerge also when an impactor is dropped into a granular bed of low density where the impactor creates a void that when it collapses causes a large but tightly confined splash to shoot upwards, see^20^,^21^,^22^,^23^,^24^,^25^,^26^ and the discussion above.

Self-organized granular jets are also observed when downhill flows of granular matter collide at the lowest point of a valley. At this point, the incoming rapid convergent flows parallel to the ground come suddenly to rest, such that the energy which cannot be dissipated through particle collisions gives rise to a vertical jet emerging from this point. An convenient experimental system characterized by persistently colliding downhill flows is a vertically vibrated short cylinder, proposed in ref. 36 where the convective flows have the required geometry. In the present paper we adopt this setup to produce sequences of granular jets, similar to the oscillon instability^35^ with two important differences: First, the novel type of jet appears in each period of the driving vibration unlike the oscillon which is a subharmonic effect of second order. Second, in contrast to the oscillon, the periodic appearance of the jet is superposed by another subharmonic instability whose order is a function of the parameters of driving, amplitude and frequency. The latter type of subharmonic oscillation is the main subject of the present paper. We observed this novel effect to occur as an subharmonic effect of up to order 11.

## Results

**Figure 1:**
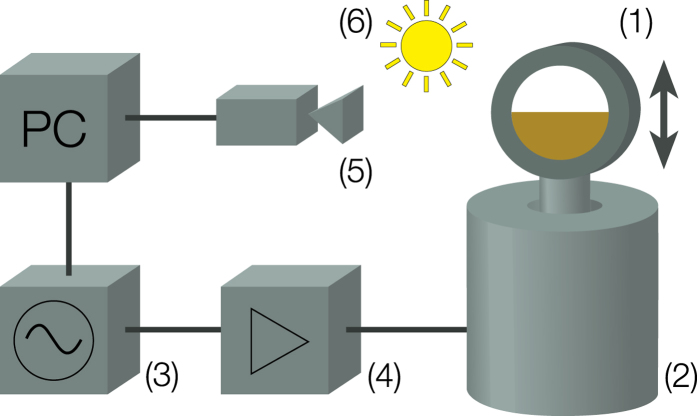
Experimental setup. A cylindrical container (1), partially filled by granular material is sinusoidally vibrated by an electromagnetic shaker (2) driven by a remote controlled function generator (3) connected to a power amplifier (4). The granular flow is recorded by a high-speed camera (5) and illuminated by an LED-Panel (6). By means of a position sensor, the amplitude of the vibration can be adjusted with high precision. Shaker, camera and illumination are controlled by a computer (PC).

**Figure 2:**
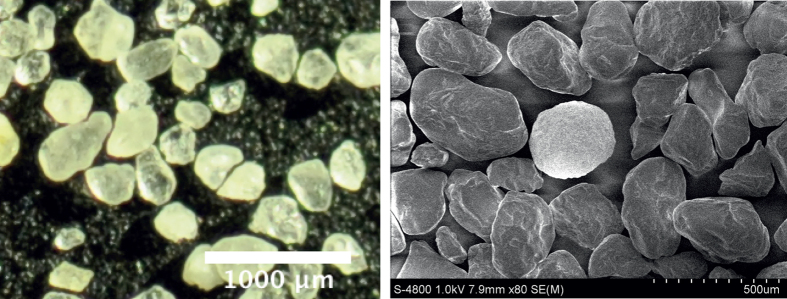
Optical (left) and SEM (right) micrographs of the used quartz sand.

The experimental setup is sketched in Fig. 1. A cylindrical container of inner diameter 12 cm and length 1 cm is half filled by bright quartz sand of approximately 300 *μ*m particle size (see Fig. 2) vibrated by an electromagnetic shaker. The granular flow is recorded by means of a high-speed camera. The results presented below are obtained from this video footage.

**Figure 3:**
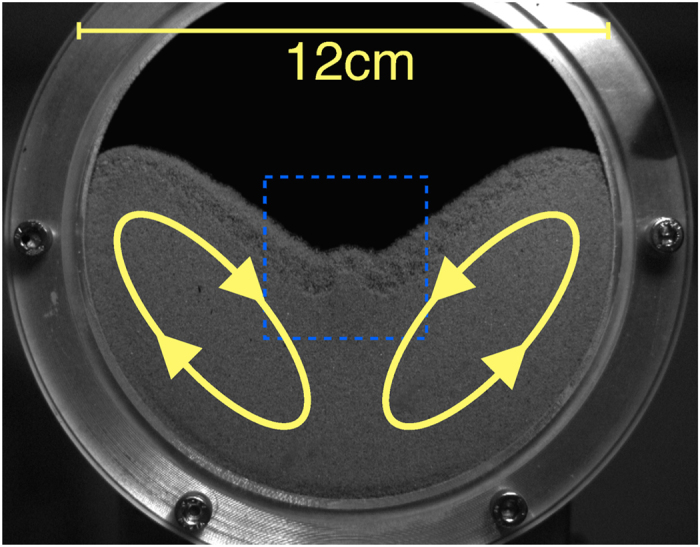
Annotated snapshot of the experiment. For sufficiently strong vibration Γ ≳ 1^37^,^38^, one observes convection^36^ as indicated by the yellow lines and a characteristic V-shaped free surface of the granulate. The corresponding downhill flow leads to a jet emerging close to the center of the system. The region of the system used for further analysis is marked by a blue line. For the figure *A* = 1.60 mm and *f* = 33 s^−1^ was chosen.

When the system is sinusoidally vibrated in vertical direction, *z* = *A* cos(2*πf*), with sufficiently large amplitude and frequency, Γ ≡ *A*(2*πf*)^2^/*g* ≳ 1, where *g* is the gravitational acceleration, one observes pronounced convection flow^36^. Figure 3 shows a snapshot of the experiment; the convection is schematically shown by superimposed yellow lines. The convective flow entails also the characteristic V-shape of the of the free surface. The corresponding downhill flows implies violent collisions of the particles at the valley where the flows clash. Here, velocity component of the particles in direction along the slopes ceases suddenly and part of the corresponding energy which is not dissipated by inelastic particle collisions, forms a vertical jet close to the center of the system.

**Figure 4:**
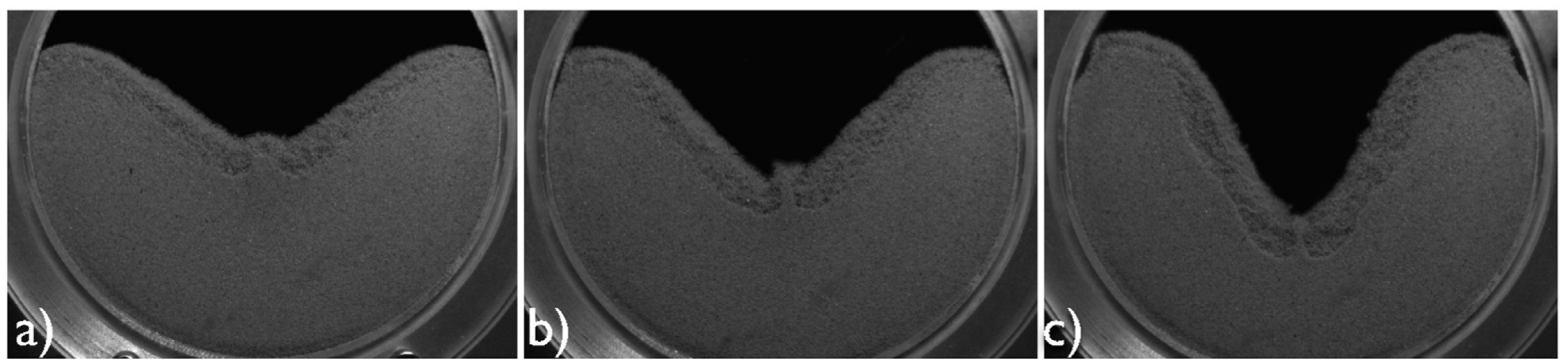
Snapshots of the system vibrated at *f* = 33 s^−1^ and amplitude *A* = (1.60, 1.98, 2.70) mm (panels (**a**–**c**)). For video sequences of the scenarios (**a**–**c**) see^39^. (**a**) shaking is sufficiently strong that a stationary jet is created by the colliding convective flows (**b**) increased shaking strength leads to the jet oscillating back and forth (**c**) even higher accelerations lead to complex behaviour of the granular flow.

From this argument it is plausible, that the size of this jet grows with the intensity of the convection which depends on the amplitude and frequency of the driving. Figure 4(a–c) shows the system at *f* = 33 s^−1^ for different values of the amplitude. We find that with increasing amplitude, the convection flow becomes more intense and the jet increases in size (a). Further increasing the amplitude, the central horizontal position of the jet becomes unstable and the jet starts travelling back and forth in horizontal position (b). For yet larger amplitude (c), the convection pattern becomes more complicated and the horizontal oscillation of the jet disappears gradually. The dynamical behavior of the system can be better seen in video sequences corresponding to the snapshots shown in Fig. 4, provided as supplementary material^39^.

**Figure 5:**
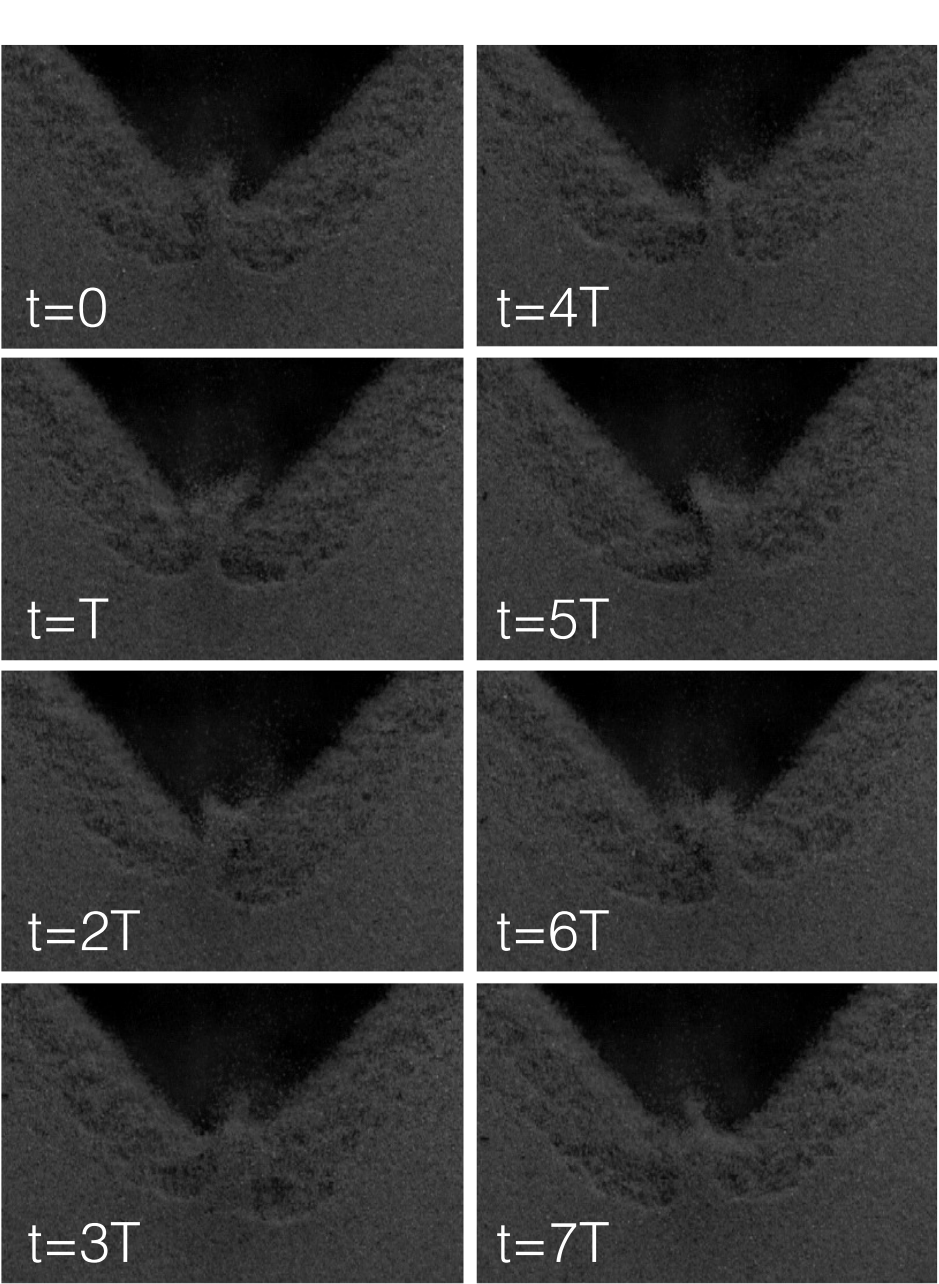
Sequence of pictures for *A* = 1.97 mm and *f* = 34 s^−1^ showing one period of the horizontal oscillation of the jet. The oscillation is subharmonic of order 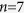. See also in the supplementary information.

Of particular interest is the intermediate regime when we observe a horizontal, oscillatory motion of the jet which we are going to characterize further. From visual inspection, we find the most salient property of this oscillation which is its stability. The oscillation is a subharmonic effect of higher order as its period, *T*_*s*_, is an integer multiple, *n*, of the periode, *T*, of the driving vibration, *n* = *T*_*s*_/*T* = *f T*_*s*_ = *n*. In our experiments we found values 6 ≤ *n* ≤ 11. Figure 5 shows a full period, *T*_*s*_, of the lateral oscillation of the jet which takes *n*/*f* = 7 · 1/34 s ≈ 0.2 s. The amplitude of the vibration is *A* = 1.97 mm. A corresponding video is provided as supplementary material^39^.

**Figure 6:**
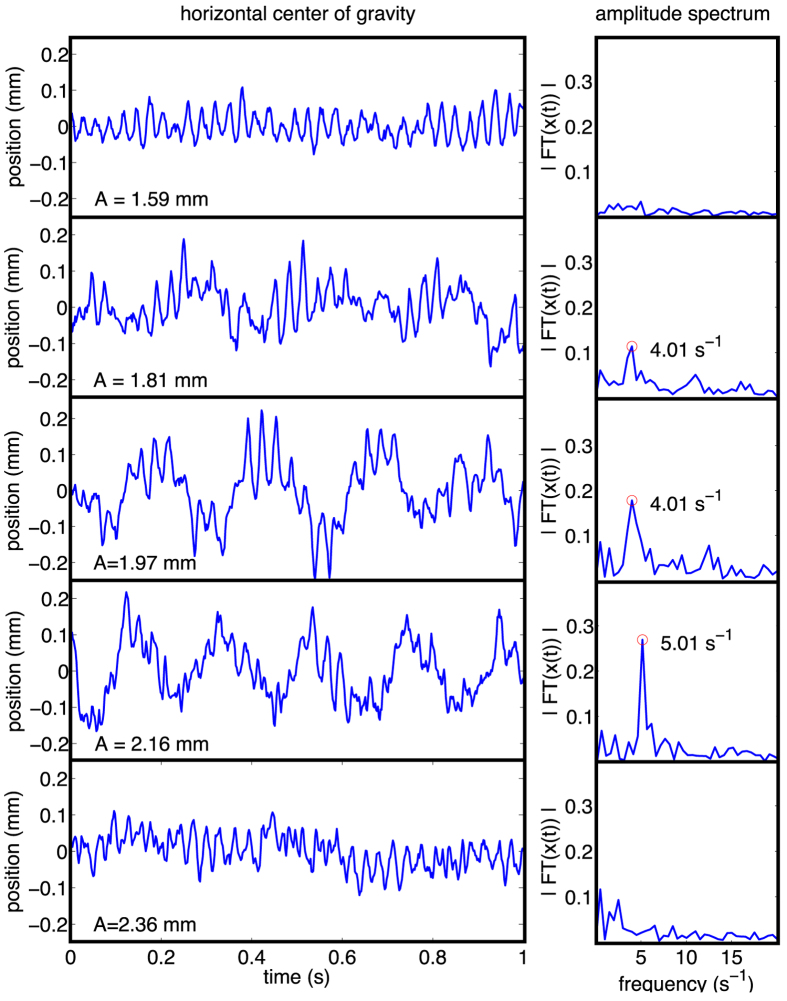
Left panels: Horizontal coordinate *x*(*t*) of the center of gravity, indicating the position of the jet, for frequency *f* = 34 s^−1^ and amplitude *A* = (1.59, 1.81, 1.97, 2.16, 2.36) mm (from top to bottom). The right panels show the corresponding absolute values of the Fourier-spectra, 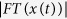. For low and high values of amplitude, the horizontal position of the jet is almost invariant in the middle of the container. For intermediate values of amplitude, the Fourier spectra evidence a pronounced and stable subharmonic oscillation.

We quantify the horizontal oscillation by means of the video footage of the high-speed recording (for details of the analysis see Sec. *Methods*). Figure 6 (left panels) shows the horizontal coordinate of the jet position as a function of time for a fixed frequency, *f* = 34 s^−1^, and several values of the amplitude of shaking. From these series we compute the corresponding Fourier spectra (right panels in Fig. 6) to determine the frequency of the oscillation of the horizontal position of the jet. From the spectra we see that for fixed frequency there is a range of amplitude where we find a stable subharmonic oscillation of the oscillation of the jet, where the order of the subharmonics depends in a systematic way on the amplitude. For small and large amplitude of vibration, the oscillation of the jet's horizontal position ceases.

**Figure 7:**
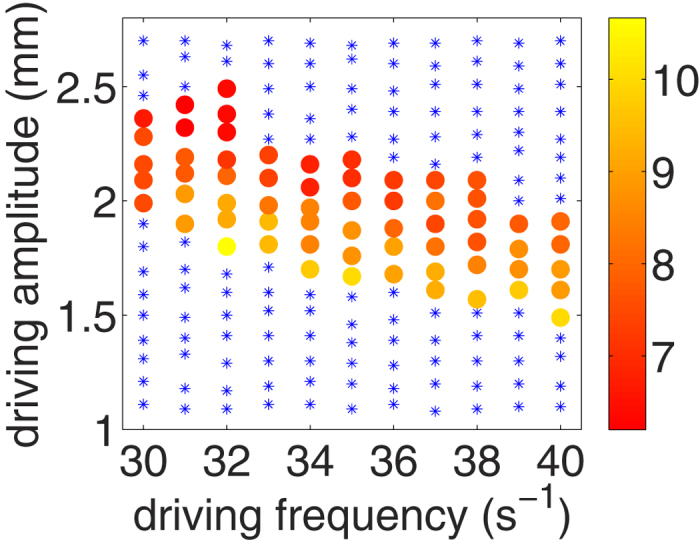
Order of the subharmonic response of the oscillation of the jet's horizontal position for a system driven at frequency *f* and amplitude *A*. The subharmonic order, *n* ≡ *T*_*s*_/*T*, appears color coded from yellow to red. Blue data points indicate parameters we were not able to distinguish a peak above the noise level in the Fourier-spectrum of the center of mass position. That is, the V-shape was clearly present, as was the jet, however it did not show regular lateral osciallation.

So far, we described the oscillation of the jet's horizontal position for fixed frequency as a function of amplitude. Obviously, the intensity of the convection is not only dependent on amplitude but also on frequency. Therefore, since the convection flow drives the mechanism leading to the jet, one may expect that the oscillation of the jet depends also on the frequency of driving. Figure 7 characterises the jet's horizontal oscillation as a function of both amplitude and frequency, where the color codes the order of the subharmonics, *n* = *T*_*s*_/*T*.

## Discussion

We consider a flat cylindrical container partly filled with granular material, with its symmetry axis oriented in horizontal direction. When this system is subjected to vertical sinusoidal vibrations, one observes a convection pattern^36^ and a characteristic V-shape of the free surface. The corresponding downhill flows which collide in the lowest point of this valley lead to a self-organized granular jet. In dependence on the parameters of the vibration, amplitude and frequency, this jet may reveal a complex temporal behaviour. In particular, for a certain range of parameters, the horizontal location of the jet follows a periodic motion which is a stable subharmonic of order *n* = 6, …, 11 to the frequency of the driving vibration, that is, the timescale of the jet's periodic oscillation is much larger than the timescale of the driving vertical oscillation. In similar experiments (not reported here) using different materials we observed up to *n* = 21.

The subharmonic oscillation of the jet's position is a novel effect which was not reported in the literature so far. The effect seems to be robust, that is, in non-systematic experiments we found it for a variety of different experimental conditions, including different container geometry (larger radius, larger height of the cylinder), different filling level, different particle size. Although subharmonic behavior is rather common for different granular systems, such as Faraday waves^39^ and many other vibro-agitated systems, e.g.^35^,^40^,^41^,^42^,^43^,^44^,^45^,^46^,^47^, we believe that the here reported novel effect is special for two reasons: first, its extraordinary high order of subharmonic response of up to 11. Second, while most subharmonic effects in granular systems are due to a synchrony between the flight time of the granulate or parts of it in the container and period of driving (see^48^,^49^ for a detailed discussion) the mechanism leading to the periodic motion of the jet's location is not so clear. In particular, the role of ambient air is still unclear. In the literature on systems rather similar to ours, some references report strong influence of air. e.g.^5^,^23^,^50^, others deny an effect of ambient air, e.g.^6^.

## Methods

### Details of the experimental setup

The cylindrical container is manufactured from aluminium with plane front side from glass coated to avoid electrostatic charging. The amplitude of the vibration imposed by the electromagnetic shaker (TIRA S 5220–120) was permanently measured using an Hall effect based position sensor^51^ with sample rate 10 kHz. This information allows to adjust the amplitude up to high precision with uncertainty ±50 *μ*m. The granular flow was observed by a high speed camera (MotionScope M3), at a frame rate 500 fps and with a spatial resolution 1024 × 1280 pixels. The camera is equipped with a *f* = 25 mm lens set to an aperture of *f*/2. For good optical contrast of the displayed images, an image of the black back wall (empty container) was subtracted from the images of the filled box. For illumination we used a LED panel. In addition to the position sensor, we checked the amplitude and frequency of the vibration from the location of a marker at the container wall which was always present in the range of vision of the high-speed camera, and could be evaluated *à posteriori*. The setup is sketched in Fig. 1.

### Analysis of the horizontal position of the jet

To quantify the time-dependent horizontal position of the jet, we analyze the frames of the high-speed video recording. First we chose an area which certainly contains the jet for the entire range of amplitude and frequency investigated. This area is marked by a blue line in Fig. 3. For each frame, we consider the field of brightness, 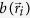, in this area. The horizontal position of the jet is found from


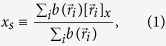


where the summation is performed over all pixels, *i*, in the marked area and 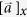 stands for the horizontal component of 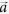. The left panels of Fig. 6 show the evolution of *x*_*s*_. The right panels of Fig. 6 are the Fourier transforms of the discrete time series, *x*_*s*_(*k*), where *k* stands for the frames of the high-speed video. The order of the subharmonics shown in Fig. 7 is then determined from strongest mode in the amplitude spectrum which is lower than the driving frequency.

## Additional Information

**How to cite this article**: Kollmer, J. E. and Pöschel, T. Subharmonic instability of a self-organized granular jet. *Sci. Rep.* **6**, 22520; doi: 10.1038/srep22520 (2016).

## Supplementary Material

Supplementary Information

Supplementary Movie S1

Supplementary Movie S2

Supplementary Movie S3

Supplementary Movie S4

Supplementary Movie S5

Supplementary Movie S6
