# Supplementary material for: Subharmonic instability of a self-organized granular jet
Source: Sci Rep. 2016 Mar 22;6:22520. doi: 10.1038/srep22520 (PMC4802379; doi:10.1038/srep22520)
Supplement: Supplementary Information [file srep22520-s1.docx]

**Subharmonic instability**

**of a self-organized granular jet**

(supplementary material)

J. E. Kollmer and T. Pöschel

**Videos of the dynamical states shown in Figure 3**

Video 1 – Titel: “f=34 Hz A=1.52 mm”, File: 34HzAcq_A_006_1.52mm.mov

Video 2– Titel: “f=34 Hz A=1.91 mm”, File: 34HzAcq_A_010_1.91mm.mov

Video 3– Titel: “f=34 Hz A=2.78 mm”, File: 34HzAcq_A_019_2.78mm.mov

For low agitation the jet is stationary (Video 1)

For stronger agitation the jet moves back and forth. (Video 2)

For even higher agitation the flow becomes more complex and the jet is stationary again. (Video 3)

**Video of horizontal oscillation of the jet,**

**corresponding to Figure 4**

Video 4– Titel: “f=34 Hz A=1.97 mm”, File: crop_34HzAcq_A_011_1.97mm.mov

The jet moves back and forth once while the container is shaken seven times. As a guide to the eye a white cross is places in the center of the frame.

**Marked version of the dynamical states video shown above**

Video 5– Titel: “f=34 Hz A=1.91 mm”, File : markers.mov

Video 6 - Titel: “f=34 Hz A=1.91 mm”, File: markers_closeup.mov

The same video as above but we now added markers to the tips of the V-shape to illustrate that we could not see any shifting of the whole granular mass inside the container, that is, the horizontal coordinate of the center of mass of the granulate is invariant up to statistical fluctuations. (Video 5). Close-up of the marked video. (Video 6)
